# Developing an understanding of coherent approaches between primary and secondary teachers: a case study within the design and technology curriculum in Scotland

**DOI:** 10.1007/s10798-022-09795-6

**Published:** 2022-12-11

**Authors:** Liza Hart-Anderson, Richard Holme

**Affiliations:** University of Dundee, Perth Road, Dundee, Scotland, UK

**Keywords:** Design Technology, Primary education, Secondary education, Curriculum coherence, Teacher CPD

## Abstract

This study is based around Education Scotland’s ambition to create a coherent learning framework for pupils aged 3–18, with particular focus on the technologies curricular area, and more specifically the subject of design and technology (D&T). The study investigates the views, definitions, and approaches adopted by primary and secondary educators applied to the D&T curricular area. Furthermore, the research explores curricular understanding and pedagogical approaches in addition to individual teacher’s understanding of technology education. A mixed method research approach was utilised and applied within one local authority region in Scotland. Data was collected from primary teachers and secondary design and technology teachers using online questionnaires and interviews. Findings reveal that there is a varied approach to teaching design and technology across primary and secondary schools with educators recognising different definitions and pedagogical approaches in the subject. This indicates that pupils transitioning from primary to secondary learning will have to cope with these differing teaching approaches when studying design and technology. However, participants agree on the importance of the design element and application of the subject to real world scenarios. It is recommended that school communities find opportunities to collaborate further with the aim of creating a more continuous, coherent learning journey for young people in the design and technology curriculum area. These findings provide a basis for future professional discussion and critical reflection for practitioners in both primary and secondary sectors, and for leaders and administrators across Scotland, the UK and around the world.

## Introduction

In Scotland, the governmental organisation responsible for education policy and curriculum places high importance on an educational experience that prepares young people for twenty-first century life and learning (Education Scotland, 2019). On a curricular or sectoral level, technology plays a vital part in future economic, societal, and environmental successes (SEEAG, 2012; Parker et al., 2021; Scottish Government, 2021). The fast-changing pace of technology indicates that Scotland’s young people must be prepared with knowledge and skills required for current and future challenges (Scottish Government, 2014) and so the subject area of Design and Technology (D&T) clearly has a far-reaching impact.

An integral guiding principle of Scotland’s statutory school age Curriculum for Excellence (CfE) is that a coherent learning experience is conducive to effective, high-quality learning. However, many researchers argue that a truly coherent experience can be challenging to achieve where several different factors can impede success (Dakers & Dow, 2009; McPhail, 2021). This is particularly true of design and technology education, where studies have shown that divergent approaches can exist between primary and secondary educators (SEEAG, 2012; Education Scotland, 2014). Furthermore, Education Scotland (the Scottish Government agency for education) found that pupils in early years and primary school settings are not receiving their full entitlement to design and technology education (Education Scotland, 2014). Although many positive examples of effective teaching were identified across Scotland, a ‘perceived complexity’ (Education Scotland, 2014, p. 41) in addition to teacher’s confidence were recognised as contributors to insufficient curriculum development occurring.

Braund (2008) has identified the importance of a coherent experience, between primary and secondary sectors, within planning and delivering curriculum. This experience is not only associated with high quality learning (Growney, 2013) but when applied inconsistently can lead to adverse effects on attainment, progression, and motivation (Braund, 2008). Therefore, research into teaching approaches within D&T, across the primary and secondary sector, is necessary to better understand and promote a key aim of Scotland’s educational framework. There are also implications for other education systems around the world.

### Research aim

This research project investigated the level of coherence within the learning framework for D&T education in Scotland. Specifically, primary and secondary teaching approaches were analysed to discover whether they align or contrast. As a result, the following overarching aim guided the research:

How do primary and secondary teachers define and approach D&T education and what are the implications of this?

## Literature review

### Coherence in education and curriculum

The issue of coherence between different phases of education has been debated by researchers for decades, possibly illustrating the challenges faced in creating and maintaining such a framework (Dakers & Dow, 2009). In their consideration of coherency, Hammerness (2006) create the concept of an ‘integrated’ (p. 1242) learning experience where acts of collaboration and unity are supportive of a connected education system. An understanding of coherency can also include attributes of ‘connection or consistency’ and can be summarised as ‘all parts or ideas fit together well so that they form a united whole’ (Collins, 2021, no page). This is particularly appropriate when considering the three main education stages in Scotland (discussed in more detail later). However, Buchmann and Floden (1992) discourage educators from using the terms coherency and consistency interchangeably, suggesting that although the two show some similarities, the differences must be clarified in the discussion of a coherent and continuous educational structure. Specifically, Buchmann and Floden (1992) warn against the risks of a consistent educational approach suggesting, ‘where a curriculum veers towards consistency, it verges towards narrowness, rigidity and the dispossession of learners’ (p. 15).

**Fig. 1:**
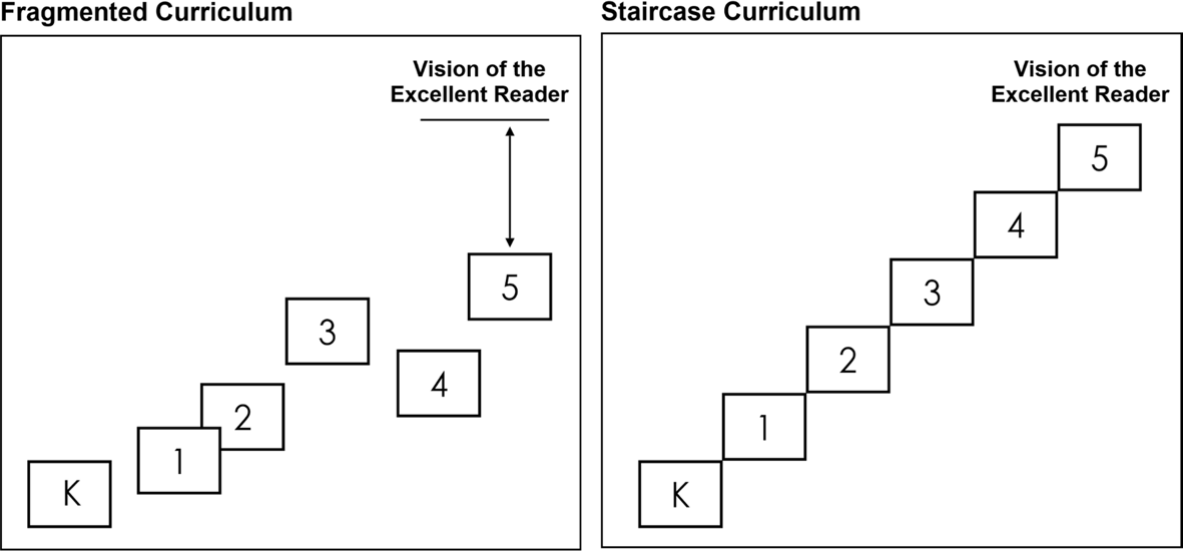
Illustration to show approaches to progressive learning

The development of a progressive learning experience requires teachers to build on previous experiences, supporting challenge and attainment, whilst avoiding repetition in learning (Green, 2020). Although designed with reference to literacy development and reading competency, Au and Raphael (2011) use the metaphor of a staircase to illustrate this progression, showing a comparison between a fragmented and coherent approach (Fig. 1). In this model, the fragmented curriculum represents discontinuities, differing approaches and gaps in learning which is depicted through fluctuation of steps and failure to meet the end vision of literacy attainment. Contrastingly, the staircase curriculum shows a continuous approach that is balanced and, fundamentally, considerate of prior and future learning (Williams, 1997). Applying this model to the primary to secondary learning progression, this concept is logistically more challenging due to change in context and teaching personnel. Within the Scottish context, a shared understanding of progression related to the curriculum framework, learning and teaching, assessment and child development is encouraged (Education Scotland, 2014).

### Coherence in the Scottish education curriculum context

The terms Curriculum for Excellence (CfE) and Broad General Education (BGE) are specific to the Scottish education context and relevant in this research project. The subsequent sections will explain and analyse these in greater detail.

### Evolution of Scottish curriculum framework

Prior to the introduction of Curriculum for Excellence (CfE), Scotland’s national education framework, was organised into three distinct stages: Curriculum Framework for Children [ages] 3–5; the [ages] 5–14 Curriculum, and the [post-14 age] National Qualifications Framework (Berry & Kidner, 2008). To create meaningful progression in a young person’s educational experience, CfE was designed around the objective of creating a coherent curriculum experience from ages 3 to 18 (Scottish Government, 2008). Furthermore, the updated narrative to supplement CfE, published in September 2019, displayed ‘curriculum coherency’ (Education Scotland, no page) as a top priority amongst curriculum entitlements for young people.

The governmental organisation Education Scotland further highlights coherence and continuity within the ‘How Good is Our School?’ documentation (Education Scotland, 2015). All schools are challenged to ask themselves: ‘To what extent do staff have opportunities to develop a shared understanding of progress in learning across levels and into the senior phase; and effective approaches to learning and teaching?’ and ‘Do we have a shared understanding of what progression looks like?’ (Education Scotland, 2015, p. 43). These questions highlight Education Scotland’s recognition of progressive learning through a coherent approach in the context of excellent practice.

### Curriculum for excellence 3–18 framework

**Fig. 2:**
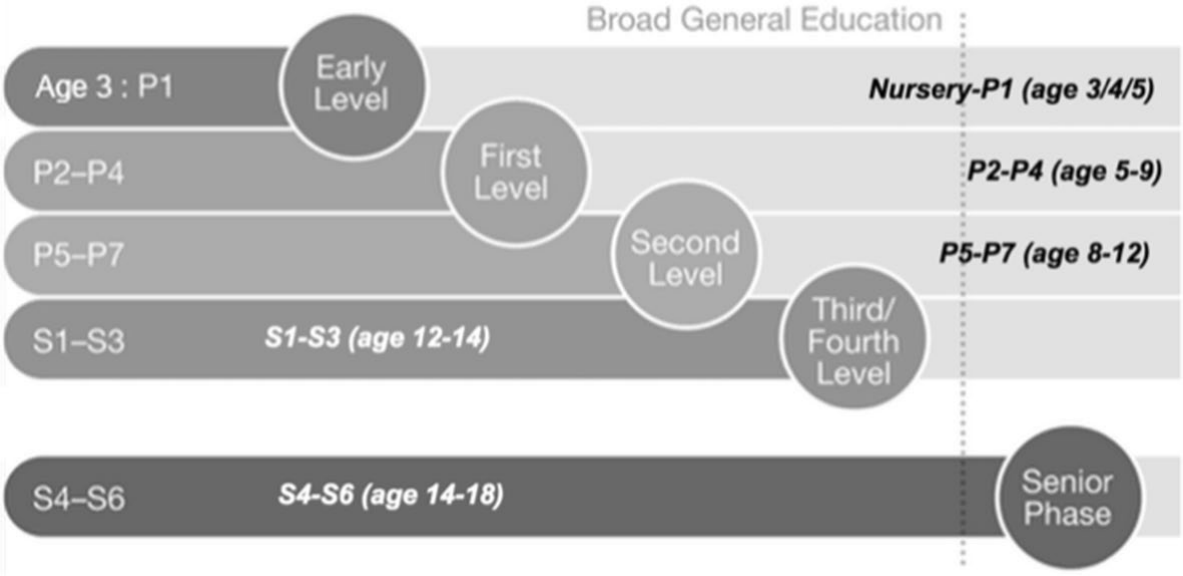
Scotland's curricular Levels

Although the curriculum in Scotland has moved away from the detached learning stages mentioned previously, the curricular framework is now split into 2 distinct learning stages: Broad General Education (BGE) from early years to approximately secondary 3 and subsequently beginning the Senior Phase in secondary 4–6 (Fig. 2, Education Scotland, 2021). To ensure coherency, all practitioners teaching within the BGE phase follow a shared curriculum framework titled ‘Experiences and Outcomes’ (Education Scotland, no date) which describe learning and progression across subject areas. The provision of this shared documentation, including prior and future learning, is considered essential in planning for a coherent and differentiated educational experience (Moore, 2008; Green, 2020). Growney (2013) notes, however, that we cannot assume educators use this information in their planning, suggesting that often educators can fail to plan far forward, or look back during their preparations. This is particularly true around the point of school transition of pupils from primary to secondary school which creates a physical barrier for primary educators to prepare students for future learning, and secondary educators to review prior learning. Transition between the BGE and Senior Phase curriculum is slightly different, where pupils are normally located in the same school, and secondary teachers tend to be directly involved in both stages so have adequate knowledge to adapt and prepare their curriculum (Growney, 2013).

### The Broad General Education (BGE) technologies curriculum

Although the school subjects of design and technology are commonplace in global, statutory education there can be differences in understanding of the terminology. This presents issues for researchers and teachers, especially where the recognised terms, and scope, may differ between primary/elementary and secondary/high school sectors. Within the Scottish secondary education sector, Design and Technology (D&T) is used widely whereas the term technology education (which features as a distinct curricular area within Curriculum for Excellence framework) is more recognisable to primary practitioners. Therefore, the use of the term Design and Technology (D&T) and technology education will be both used in this paper depending on the specific context.

The CfE Technologies curriculum, at BGE level, constitutes one of eight curricular subject areas which collectively are identified as a core focus of the curriculum (Education Scotland, no date). The five levels of BGE progress from early level (youngest learners) through to National Qualifications during the Senior Phase. Learning aims for each level are outlined in the *Technologies Experiences and Outcomes* and *Technologies Benchmarks* documentation (Education Scotland, 2017).

Within the Senior Phase, young people are then streamlined into separate technology subjects, for example Graphic Communication or Computing Science, which are organised by the Scottish Qualifications Authority (SQA, 2021). The technology subjects offered vary on a local level dependent on availability of resources, including staffing and school level departmental organisation.

The technologies curricular area includes food and textile technology; technological developments in society and business; craft, design, engineering; and graphics and computing science. Anecdotal evidence suggests secondary D&T departments will focus on the craft, design, engineering and graphics elements of the technologies curriculum. In contrast the specialist secondary practitioners are trained and qualified in slightly different subject specialties such as practical wood working and graphic design offered by Scottish Higher Education Institutions (The University of Edinburgh, 2021; University of Glasgow, 2021; University of the Highlands and Island, 2021). This inconsistency was highlighted by Education Scotland’s (2014) study into the technologies curriculum which determined that the technologies ‘brand’ (p. 41) needed to be clearer. This issue mirrors Dakers’ (2006) conclusion suggesting that establishing continuity of learning in D&T has made little progress in the intervening 15 years.

Clearly there are external challenges that impact how effectively D&T is delivered in both primary and secondary sectors which provides further justification for the research reported in this paper.

### Shared understanding of D&T education

The challenge of achieving a coherent experience across ages and stages within D&T teaching may be due to differences in the way teachers from the primary and secondary sectors understand and interpret the nature of the subject. Braund (2008) is clear that academic coherency must imply ‘a consistency of aims, values and expectations’ (p. 6), further highlighting a need to look beyond the written curriculum guidance. Many authors (e.g. Ely, 1983; Jones, 1997; Hammerness, 2006; Rohaan, Taconis and Jochems, 2010; Wellcome Trust, 2014; Gill, 2018) consider that underlying principles, as well as knowledge, skills and terminology, should filter down and directly impact on teaching. Furthermore, Moreland, Jones and Northover (2001) argue that teacher beliefs about technology education are influential on their practice whilst Ely (1983) proposes that practitioner’s teaching decisions and classroom practice are shaped by their own personal definitions of a subject. Where practitioners’ definitions are based on differing values and understanding there is the potential for disjointed, incoherent learning experiences, not only between different schools but also between teachers within the same school.

### Individual teacher understanding of D&T education

Lane (2019) notes that different individuals or groups may distinguish school subjects based upon their own experiences. This too has been considered in the educational field with Zanker and Owen-Jackson (2013) indicating that educators may base their planning and teaching of a subject on their own personal experience and expertise. A fixed definition of technology education that does not evolve in line with societal and educational priorities can result in problems for both teachers and learners. As well as causing confusion for practitioners, this could also be damaging for the progression of the subject on the whole (Rohaan, Taconis and Jochems, 2010). Most importantly, a disparate understanding can also cause issues when endeavouring to create a continuous approach between school stages, as discussed earlier.

### Nature of D&T education in policy

Through Education Scotland’s 2014 study, they proposed that creativity along with problem solving, in the context of the real world, should be distinguished as the ‘core business’ (p. 36) of technology education. Bowen (1996) also recognises the development of creative problem-solving skills in technology education through the blend of knowledge based learning and practical activities. The intention of Scottish Universities to develop the teaching workforce (University of Edinburgh, 2021) supports this objective whilst also highlighting the underpinning technical foundations, describing technology education as:

developing technological capability through the combination of designing and making skills with technological knowledge and understanding of values, consequences and bigger issues of technology in society and sustainable development (University of Edinburgh, 2021, no page).

This illustrates the importance of Design and Technology education to wider society, and both primary and secondary sectors have a role to play in developing this. This adds further weight to the importance of investigating this shared understanding between different sector practitioners.

### Teacher approaches to D&T education

As this research project focuses on teachers’ definitions of, and approaches to, D&T teaching it is important to consider issues relating to delivery of teaching, and how these impact on learners. Braund’s (2008) study, which focusses on progression and continuity in science learning, discovered that discontinuities between stages of schooling can cause academic disruption and, at times, regression in the learning of young people. Braund’s (2008) findings concentrated on three distinct areas of continuity to support academic progression: curriculum, pedagogy and assessment. As three key areas of learning that appear to be overlooked in D&T research, these will be important when framing discussion of results from this project. Examination of these areas provides a background to the understanding of a coherent teaching approach as well as the potential influence that academic discontinuities and disruption can have.

Curriculum, pedagogy and assessment are often perceived as the fundamentals of teaching and learning where each element is an influential determinant of the learning experience a young person will receive (Devasagayam and Mahaffey, 2008). Indeed, these educational themes are highly complex (De Rossi and Trevisan, 2018), challenging educators to spend their whole career transforming their approach and striving for continued excellence. In their most basic form however, the curriculum is an agreement of what is to be learned; pedagogy incorporates how this knowledge will be delivered to pupils and assessment aims to understand what students know and in turn, what will happen next. These three elements are perceived to be interrelated whereupon each has an effect on the other, together creating the core of learning and teaching (Hayward et al., 2016). Crucially, this does not mean that all educators have to teach the exact same thing in the exact same way, without any flexibility or scope for personalisation. Rather, curriculum, pedagogical and assessment experiences should gradually evolve and progress through activities that support continuous educational development (Devasagayam and Mahaffey, 2008).

In the discussion of learning and teaching in twenty-first century education, all practitioners should be aiming to deliver high quality, effective learning experiences for young people (Donaldson, 2010). Curriculum, pedagogy and assessment can be considered as the foundations of these experiences which has been recognised by several authors as Pedagogical Content Knowledge (PCK) (De Miranda, 2008; Park and Oliver, 2008; Alonzo, Kobarg and Seidel, 2012; Loughran, Berry and Mulhall, 2012; De Rossi and Trevisan, 2018). This complex combination of knowledge and skills is considered to encompass differing strands of learning where an insufficiency in any strand has been recognised as a limitation (Johnston and Ahtee, 2006). Within their research into science PCK, Park and Oliver (2008) identify 5 different knowledge concepts including: ‘orientations to science teaching, knowledge of student’s understanding, knowledge of the curriculum, knowledge of instructional strategies and knowledge of assessment of science learning’ (p. 264). Although not focussing on PCK, Growney (2013) too highlights different aspects of effective teaching that echoes Park and Oliver (2008), suggesting that educators need ‘full proficiency in the subject, that is, confidence in the knowledge, understanding and skills both pedagogic and practical, that define the identity and nature of the subject and competence to convey it through their teaching’ (pg. 53). Both Growney’s (2013) and Park and Oliver’s (2008) concepts of effective teaching suggest and emphasise knowledge out with the realms that solely the curriculum guidance has the ability to provide.

In their study into technology education in primary schools, Moreland, Jones and Northover (2001) concluded that a strong teacher knowledge base is critical in the realisation of proficient technology education where uncertainty leads to limited teaching methods. In addition, Park and Oliver (2008) directly relate teacher knowledge to efficacy. Alongside this strong knowledge, Growney (2013), Dakers & Dow (2009) assert the importance of confidence in delivery resulting in effective or restricted pedagogical and assessment approaches. Considering the multitude of strands that contribute towards effective D&T learning supports (Education Scotland’s 2014) study into the aversion that some educators feel towards approaching the subject. For primary educators, this is amplified by an expectation to apply this in a broad range of curriculum areas.

### Conclusion to literature review

The curriculum subject of Design and Technology is interpreted in a variety of ways by teachers, leaders, and policy makers presenting challenges in creating and applying a coherent and continuous learning framework. Inconsistent experiences in these elements have been found to result in adverse effects on progression, motivation and therefore attainment (Braund, 2008). Having established these issues this paper will detail the original research that explored different perspectives of primary and secondary educators within one local authority in Scotland exploring how primary or secondary teachers’ definitions of, and approaches to, D&T align or contrast.

## Methodology

The aim of the research project was to explore primary and secondary teachers’ ideas of design and technology education. It specifically focussed on the school phase or stage referred to as Broad General Education (BGE). The main aim and research question was:

How do primary and secondary teachers define and approach D&T education and what are the implications of this?

All research conducted was driven by the principles of the pragmatic paradigm which places the question at the forefront of research (Clarke and Visser, 2018). Above all, and fundamental to the success of this study, were the ethical principles and standards established by the host academic institution (University of Dundee, 2016). All research performed was therefore approved in advance by the university’s ethical governing body.

### Justification for mixed method approaches to research

Many researchers discuss the mixed method approach as an effective route to gather social research (Fossey et al., 2002; Bryman, 2006; Creswell and Garrett, 2008; Komorowska, 2016; Boeren, 2018). Increased knowledge of a subject plus a deeper understanding has led to several researchers becoming advocates of the approach (Creswell and Plano-Clark, 2007; Glogowska, 2011). However, the mixed methods approach carries many cautions, specifically from those whose research principles originate from differing ontological and epistemological values (Cleland, 2015). In addition, Bryman (2006) argues that when research methods are combined, the researcher can be led to unanticipated results. In this case the researcher and co-author acknowledge their epistemic and ontological influences, and where they may be drawn more to positivist methods, and therefore considered this during the analysis and discussion stages.

The research design consisted of two research stages to create a mixed method triangulated study: namely quantitative self-completion, online questionnaires, and qualitative interviews. Denscombe (2014) discusses the advantages of triangulated studies suggesting that there is the potential for increased validation by interpreting findings from differing outlooks and corroborating data from multiple sources. It was anticipated that adopting a quantitative research approach would gain a broader understanding of the subject reaching across the local authority whilst a qualitative method would provide deeper contextualised information to further comprehend and justify these findings (Fossey et al., 2002; Ercikan and Roth, 2006). Therefore, the research project included two stages, questionnaires at Stage 1 and interviews at Stage 2.

### Stage 1-Questionnaire sample

Although the location and specific policy context for this project is Scotland, the topic is relevant to school systems that feature D&T around the world. Limitations of available practical resources determined the extent of the research sample and so the decision was made to focus data gathering on one local authority area within Scotland. This was further justified as, although differences exist between local authority areas, the Broad General Education and Curriculum for Excellence framework are consistent curriculum structures across the statutory education sector in Scotland.

When considering sample sizes, Gorard (2003) encourages quantitative researchers to be ambitious by developing a larger sample size. By developing a larger sample size there is an expectation that there will be a greater number of responses which can equate to findings that have the potential to be more representative of a target population (Cohen, Manion and Morrison, 2017). The online questionnaire employed a purposive sample (Sarantakos, 2013) and was consequently distributed by email to all relevant primary and secondary practitioners within the local authority. Two questionnaires were developed and sent to primary and secondary educators respectively. Both questionnaires included the same question focus however were worded using sector specific terminology to ensure relevance for their intended audience (Cohen, Manion and Morrison, 2017). This approach was used to encourage participation, reduce potential for misunderstanding, to allow for a more authentic representation of participant views, and lead to greater accuracy with analysis of data.

The purposive sampling approach consisted of primary and secondary educators who were currently teaching or who had recently taught the technologies curriculum within BGE. Following informal consultation with practising teachers, an assumption was made that all secondary D&T teachers would currently or have recently taught the BGE D&T. Consequently, all secondary D&T teachers in the target population were invited to take part (n = 74).

The variable structures of primary schools within the local authority and primary educators’ potential annual movement between teaching year groups resulted in a sampling framework that was challenging to define. For purposes of efficiency, all primary school headteachers in the target local authority area were contacted with a request that questionnaires were passed to all primary teachers, within their setting, who met the required criteria (n = 176).

Although a larger number of respondents were contacted through this study (total available sample n = 250), the actual number of participants that responded could not be taken as being perfectly representative of the teaching community in the local authority. Therefore, it was accepted from the outset of the study that a definitive conclusion would not be possible. Nevertheless, the data gathered, and subsequent analysis and discussion provide topics that practitioners, leaders, and administrators can use as a basis for reflection and professional discussion in future.

### Stage 1-Questionnaire design

The questionnaire began with an open question asking participants to give their definition of technology education, acting as an introduction to the theme of the research. Sarantakos (2013) considers the richness of data that can be generated through this approach where alternative, pre-set criteria would have undoubtedly influenced statements and potentially limited genuine or authentic responses. Furthermore, using a closed question may have influenced the responses that could be interpreted as less reliable as respondents are forced to give an answer that may not accurately represent nuance in their views (Taylor and Medina, 2011).

Data gathered from the open question was coded allowing the ease of analysis in the later project stages. Key terminology from the literature review related to technology education was used as a reference point. Other interesting terminology and words that participants responded with were also added to the list. Each response was then checked against the list of key terminology allowing a frequency of data responses to be compiled.

The remainder of the questionnaire involved closed questions utilising a combination of Likert type-scales and dichotomous choice questions. Dichotomous questions were used as a method to gain clear, definite answers regarding collaboration activities across the primary and secondary stages. These questions were supplemented with information from the interviews which Bell and Waters (2018) suggest can ‘put flesh on the bones of survey responses’ (p. 235). Likert scale questions sought to find out how often a pupil activity related to the teaching and learning of D&T education was undertaken. Respondents had the option to choose one answer, either: ‘*a lot’, ‘sometimes’* or *‘not at all’* in response to the questions. Without some form of structure, the data collected could have varied greatly and posed unnecessary challenges in the data analysis stage of research, potentially wasting time and effort. These question types were favoured to allow for accurate correlations and therefore support a simpler data analysis (Sarantakos, 2013).

### Questionnaire reliability and validity

To develop a research plan that was valid, the questionnaire design was initially piloted amongst five teaching and research colleagues. Interaction and feedback with the questionnaire were analysed and discussed between the researcher and their mentor and thus changes were made to develop a more effective research framework. The involvement of experienced academic research staff in the design process ensured questions were valid and addressed the research aim and question. Once the data was collected and analysed the same staff were involved in checking the codes and themes to reduce potential for researcher bias and enhance reliability and therefore rigour. These same underlying principles were applied to the interview design and data analysis. This is explored in greater detail in the subsequent section titled *Validity, reliability, and limitations.*

### Stage 2–Interview design

Standardised open-ended interviews were conducted to collect data at stage 2. The content and sequence of questions were organised and planned, resulting in an expansion of the comparability of responses within the data analysis stage of research. Additionally, the questions were consistent across all the interviews to maintain a uniform approach to aid with the reliability of data (Sarantakos, 2013). Initially, a face-to-face approach had been the preferred method however to offer options and encourage participation, an online video call approach was also offered. When the Covid-19 pandemic restricted social interactions, online interviews became the sole interview approach.

### Stage 2-Interview sample

The sample frame for the interviews remained the same; secondary teachers who taught D&T and primary teachers who were currently or who had recently taught primary 5, 6 or 7. Through the questionnaires, participants were invited to volunteer to take part in an interview. Ideally the sample would correlate with factors such as homogeneity of the target population, time restrictions and the quantity of data desired (Sarantakos, 2013). Assessing these factors, the decision was to have a target sample of six teachers that would include 3 primary teachers and 3 secondary teachers for even comparison. Ultimately, five teachers volunteered to participate: 3 secondary and 2 primary teachers.

### Stage 2-Interview transcription and coding

Each interview was audio recorded to allow the researcher to focus on the dialogue (Denscombe, 2014). These audio recordings were then transcribed to allow for comparison later in the data analysis process.

A combination of deductive and inductive coding analysis was used to discover keywords and themes related to technology education (Cohen et al., 2017). Initially, key terminology discussed throughout the literature was used as a foundation as well as recurring themes which emerged from the research itself. For example, the secondary school educator’s response that stated:

…technology education is a creative subject where pupils learn to solve problems in relation to real world social issues…

Which was coded under ‘problem solving’, ‘society’ and ‘creativity’. Each of these codes had already been identified through the literature review.

In total, 22 secondary and 19 primary teachers responded to the questionnaires. In addition, five participants volunteered to be interviewed at stage 2 and this relatively small overall sample meant that generalisation of results should be considered carefully (Bell and Waters, 2018) and this will be discussed later.

### Ethical considerations and informed consent

To enable potential participants to make an informed decision regarding their participation, reach-out emails that included participant information sheets and consent forms were emailed. These documents were essential in communicating key details of the study including their freedom to volunteer and their right to confidentiality. The reach-out email was particularly important as it explained the researcher’s position as a teacher and researcher therefore becoming more relatable and personable to prospective respondents (Sarantakos, 2013). Given that the participants were competent adults, without a power imbalance between them and the researcher, and data was completely anonymised the ethical considerations for this research project was deemed to be low risk. Ethical consent was granted by the host academic institution.

### Validity, reliability, and limitations

Using a triangulated mixed methods study, the validity of the data collected has been enhanced and strengthened (Denscombe, 2014). By employing both questionnaires and interviews, the data could be triangulated offering greater insight and providing a chance to corroborate common findings. Furthermore, all data was thoroughly checked for mistakes, direct quotations were embedded into discussion where appropriate and transcripts sent to interview participants to check validity (Denscombe, 2014). The researcher was conscious of potential bias during all stages of research making a conscious effort to study in a detached manner. An ‘open-minded’ (Denscombe, 2014, p. 302) approach is encouraged which was realised through the researcher’s genuine curiosity about the subject and awareness that there is nothing to gain by adding to the ‘primary versus secondary’ agenda. These steps support the validity of findings within this research project.

To develop a reliable study, guidance from literature was adhered to when designing methods, instructions used by participants were written clearly and all research decisions explained and justified within this chapter. Furthermore, the same questions were asked to all participating teachers in questionnaires and interviews providing a consistent approach. One consideration, regarding reliability, is that the stage 2 data was interpreted by a single researcher who may have been influenced by their background in secondary education, or their individual ontological or epistemic beliefs. To mitigate this there were discussions of interpretation of data between the researcher and other suitably experienced, academic peers.

Following the collection and analysis of data some limitations were identified. To enable a wider response, a large sample size was selected and contacted. The response rate was reasonable, especially given a recognised lower response in online surveys, also impacted by the fact that those who work in education may regularly be invited to participate in surveys (Kennedy and Archambault, 2012).

Due to the number of participants responding it is acknowledged the results are not representative of the teaching community across the entire local authority area, therefore a definitive conclusion cannot be drawn. This relatively small overall sample meant that generalisation of results should be considered carefully (Bell and Waters, 2018). Nevertheless, the findings pose ideas that could be used as the basis for professional discussion and self-reflection within more localised associated school groups as a way of considering coherency in D&T, the technologies as a whole and other subject areas and this will be explored in the discussion section.

As examined through the literature review, D&T is a part of the larger technologies curricular area, though the objectives of this study focus on D&T. This could have been communicated in a more obvious manner as some participants discussed aspects of the technology curriculum rather than D&T. Despite this limitation, the reference to issues such of digital technology by participants provided valuable contextual evidence for the later discussions and implications.

## Findings

The findings from the Stage 1 questionnaire are presented below in Tables 1, 2 and 3. The data from the Stage 1 questionnaire (including the initial open-ended question which considered definition of technology education) are discussed under broad thematic headings relating to: Teacher understanding of D&T/Technology education, Specialist nature of D&T curriculum, Shared Understanding of D&T concepts, Topic teaching around impact on society and application of engineering, Practical and technological skills, Teaching use of technology versus understanding technology, and Primary and secondary educator knowledge of other sector and stage of education. Where appropriate, extracts from the Stage 2 interviews have been used to triangulate the findings, by illustrating specific points, and providing greater depth to discussions. The data tables are presented where they are first relevant for the theme being discussed.

Table 1 shows primary and secondary educator’s defining themes of technology education and the frequency in which they were communicated. Following this, Table 2 displays how frequently educators delivered learning related to the ‘Craft, Design Engineering & Graphics’ (Education Scotland, no date) specific outcomes of the BGE Technologies curriculum. Additionally, the frequency in which pedagogical teaching strategies were implemented to deliver technology learning is detailed in Table 3. These findings provide an insight into primary and secondary educators’ understanding of technology education and subsequently how they approach the planning and delivery of technology learning. In understanding primary and secondary approaches to technology education, these findings allow for a comparison between learning experiences and thus supports an understanding as to what extent a coherent learning experience may exist.

**Table 1:** Primary and secondary teachers’ definitions of D&T/Technology Education

Code	Frequency (primary participants)	Frequency (secondary participants)	Total
[Impact on] society	5	9	14
Designing	7	6	13
Problem solving	3	8	11
Technological knowledge	3	7	10
Use of technology [to support learning]	9	1	10
Practical application of knowledge	3	5	8
Technological skills	2	5	7
Practical skills	2	2	4
Food & textiles	4	0	4
Creativity	0	4	4
References to technology specific subjects	0	4	4
Examples of technology	2	1	3
STEM	1	1	2
References to other subjects	1	1	2
Digital literacy	1	0	1

**Table 2:** Frequency of curricular areas taught (stage 1 likert questionnaire data)

	Often		Sometimes		Not at all	
CfE design & technology organisers (subject areas)	Primary	Secondary	Primary	Secondary	Primary	Secondary
Design and construct models	16% (3)	68% (15)	63% (12)	27% (6)	21% (4)	5% (1)
Exploring uses of different materials	10% (2)	50% (11)	74% (14)	50% (11)	16% (3)	0
Representing ideas, concepts and products through a variety of graphic media	16% (3)	64% (14)	63% (12)	36% (8)	21% (4)	0
Application of engineering	5% (1)	27% (6)	42% (8)	55% (12)	53% (10)	18% (4)

Respondent frequency in brackets. Percentage decimals rounded to the nearest whole number.

CfE Design & Technology Organisers can be found here: https://education.gov.scot/Documents/Technologies-es-os.pdf

**Table 3:** Frequency of pedagogical approaches applied (stage 1 likert questionnaire data)

	Often		Sometimes		Not at all	
Common pedagogical approaches	Primary	Secondary	Primary	Secondary	Primary	Secondary
Project based work	32% (6)	76% (16)	58% (11)	24% (5)	10% (2)	0
Topics involving real world scenarios and society	42% (8)	48% (10)	53% (10)	52% (11)	5% (1)	0
Inquiry based learning – research and investigation	11% (2)	41% (9)	63% (12)	59% (13)	26% (5)	0
Practical manufacture skills	6% (1)	86% (19)	47% (9)	14% (3)	47% (9)	0
Problem solving	58% (11)	64% (14)	42% (8)	36% (8)	0	0
Use of ICT to enhance learning	58% (11)	68% (15)	37% (7)	32% (7)	5% (1)	0
Use of other technologies to enhance learning	32% (6)	43% (9)	42% (8)	57% (12)	26% (5)	0
Use of online resources to enhance learning	37% (7)	50% (11)	53% (10)	50% (11)	10% (2)	0

Respondent frequency in brackets. Percentage decimals rounded to the nearest whole number.

## Discussion of findings

### Teacher understanding of D&T/Technology education

The main finding from this study was that the primary and secondary educators did not share a collective definition of technology education. This conclusion does not suggest that one group is ‘right’ and another ‘wrong’ but what is notable is the difference and highlights that coherence of educational experience is an issue for young learners studying in Scotland. More specifically it draws attention to the disparity amongst perceptions and understanding of technology education, the results of which have been determined to guide practitioner’s decision making in the classroom (Ely, 1983; Moreland, Jones and Northover, 2001). Primary educators more frequently affiliated their understanding of technology education with the *use* of technology, though not individual disciplines within the D&T curriculum area. For example, one primary teacher’s response to the question ‘How would you define technology education?’ was:

a valuable tool that can be used to enhance education in many positive waysif used appropriately.

Another suggested it was:

using digital technology to aid learning…

but also added that:

Technology education is about making stuff and exploring problem…

Some primary responses referred to other elements of the curriculum whilst others responded solely about the use of technology in teaching and learning. In comparison, secondary educators made more references to problem solving, the influence of society, practical skills, and specific disciplines within the D&T subjects (Table 1) and very little (1 response) to its *use* in the classroom. To illustrate this a secondary teacher stated:

Technology education is learning about technology. Technology can encompass a wide variety of study areas, from IT, electronics, using various tools and machinery, applying various approaches to solve problems, sketching, drawing, CAD/CAM. Technology Education is about stimulating a child’s creativity and about testing their inventiveness. It’s about experiencing the joy and satisfaction of making something. (Secondary teacher)

This particular response is interesting as it draws attention to the broad nature of the technologies subject under which both primary and secondary educators are expected and should be prepared to teach (Donaldson, 2010). Further, it is interesting when considering the challenges of creating a coherent learning by drawing attention to the wide range of experiences and knowledge offered.

The higher frequency, by primary teachers, of reference to themes such as *using* technology in contrast to distinct subjects in secondary perhaps indicates that technology experiences are being approached in a different manner between the two sectors. In particular, the focus from primary teachers appears to suggest that the boundaries, for example, of digital technologies and D&T have become unclear. This is notable as a common criticism of Curriculum for Excellence, especially in the primary sector, is that the guidance for teachers is vague (Priestley and Minty, 2013).

It is widely accepted that digital skills are incredibly powerful in a young person’s learning development, however this interpretation may impact, possibly diluting, more focussed D&T and technology education experiences. Furthermore, it signifies that in some circumstances, learners may only be beginning their D&T experience at secondary level, therefore the discussion of continuity and progression between primary and secondary becomes far less relevant. The makeup of the subject means that naturally, educational areas will overlap, but clarification and alignment of outcome is necessary for all educators (Rohaan et al., 2010). Therefore, addressing this issue will enable educators to ensure that learners are receiving shared concepts of D&T that have begun in primary school then continued and developed through secondary (Braund, 2008).

### Specialist nature of D&T curriculum

The differences in understanding of D&T as a subject was highlighted by two codes in the Stage 1 open-ended question (Table 1). Several primary participants (n = 4) referred to ‘food and textiles’, and the same number of secondary participants referenced ‘creativity’. Given the small sample size this may not be particularly notable but could simply acknowledge the broader interpretation of the D&T curriculum area within the primary sector. Whereas in secondary, food and textiles may be more likely to be incorporated in a subject such as Home Economics. The code of ‘creativity’ is more interesting and the lack of recognition from primary teachers may be due to the way in which D&T is seen as being more technical than other primary subjects such as those within the Expressive Arts curricular area, which incorporates art, drama, and dance.

The idea of D&T as a being either a specialist or multifaceted field also differs between sectors and individual teachers. To qualify to teach in Scotland, secondary level D&T educators are required to study in a field related to technology then specialise in subject specific teacher training (The University of Edinburgh, 2021; University of Glasgow, 2021; University of Highlands and Island, 2021). Understandably, this depth or specialism is not replicated in primary degree programmes (Gill, 2018) which prepare educators to teach and adapt to a potentially expanding curriculum and new educational initiatives. As the Donaldson (2010) report suggests, primary teachers cannot be experts in all curricular areas but must have enough knowledge and pedagogical skill to expand pupils’ learning. To improve learning and teaching, across D&T education, there must be a renewed focus on teacher knowledge and its effect on related pedagogies (Moreland, Jones and Northover, 2001). Requirements of a strong knowledge base and skill become increasingly valuable in D&T as Bowen (1996) advises teachers do not have to have ‘the answer’ (p. 15), but to see their role as facilitating children’s learning particularly where creativity could lead learning in several directions. SEEAG (2012), who are advocates for science learning, argue that primary teachers should have additional qualification requirements whilst pushing for at least 15 h of STEM professional development each year (p. 24). However, responsibility cannot be placed solely on primary practitioners, and all stages must aim to be proactive in effecting change (Donaldson, 2010) and supporting each other to do so.

To further analyse the collective understanding of the D&T curriculum, participants were asked how often they engaged with the D&T specific Technology Experiences and Outcomes (E&Os). The data (Table 2) reveals secondary educators employed these D&T outcomes much more regularly than their primary counterparts, however, this may be unsurprising given the greater opportunity and expertise with the secondary school stage. Primary educators predominantly expressed that they ‘*sometimes’* engaged with relevant outcomes. Emergent data from the interviews builds a fuller picture of this experience suggesting that, at times, in primary sector the technology outcomes can often be approached as an add-on to larger interdisciplinary projects. For example, one primary teacher states:

So, most of the technology outcomes in Curriculum for Excellence I’d probably be using the ones for digital literacy and that would be although not be stand alone and would kind of tie in with a lot of topic work that we did. So, for example our topic work right now is France and there would be digital literacy work in terms of using the laptops. (Primary teacher)

In addition, the figures reveal many primary educators do not incorporate the relevant BGE technology outcomes at all. Stage 1 data shows that 21% of primary participants do not engage in E&Os that cover ‘design and construct models’ or ‘representation of ideas through graphic media’ experiences. Additionally, 16% stated they do not address the E&O ‘explore the uses of materials.’ One primary participant responded that they do not teach any of the D&T curricular subjects at all, whilst a quarter of primary practitioners (26%) responded that they did not teach two or more of the relevant D&T Experiences and Outcomes. Overall, this data shows a clear disparity between the sectors, and what they understand, or more crucially what they are teaching within the D&T curricular area.

### Shared understanding of D&T concepts - designing and problem solving

Despite the differences between sectors there were some areas on commonality. The data from the initial open-ended definition question included a high number of references to ‘designing’ (Table 1), from both primary and secondary practitioners, highlighting that this significant element of the field is recognised across both stages. For example, one primary teacher defining this as:

being able to use knowledge, skills and understanding related to the designing, making, testing, and utilising of products (physical and digital), drawing on problem solving, researching, analysis and communication skills throughout the process. (Primary teacher)

Additional quantitative data, shown in Table 2, also shows that most participants, both primary and secondary, (88%) are engaging in some manner with the relevant design BGE technology experiences and outcomes. The design element of D&T incorporates many different strands including problem solving and creativity which Education Scotland (2014) encourages through a real-world approach. The concept of design as an element of D&T was further supported by the qualitative data, from the Stage 2 interviews where one primary teacher pointed out their concerns:

I could tell children what I would do to design something, but I am not a designer. So, it’s not consistent; I feel that somebody else could say something totally different. (Primary teacher)

Furthermore, when considering how they apply the design stage to a practical task or activity, the primary teacher further explained:

What tools and equipment [are at our disposal] and we don’t have a lot of the tools and equipment that you would have in secondary. Use a range of methods to join and strengthen materials – I could tell you right now I don’t even know what the methods are, welding? Would you weld something? (Primary teacher)

Although using similar terminology, the primary teacher clearly articulates uncertainty with their own knowledge and the curricular guidance provided by Education Scotland (2017). This is a clear example of where shared understanding of, and confidence in, D&T teaching may begin to diverge. To link back, at secondary level, the necessary ‘tools and equipment’ and ‘processes of designing’, for example, are clearly described by the SQA (2021) through the guidance put in place for Senior level National Qualifications. This guidance ultimately influences how secondary practitioners’ structure and plan for S1 to S3 BGE learning. Without the same guidance at primary level however, practitioners are left to interpret and create their own understanding.

In this situation, teachers may therefore have to rely on their own personal experiences of technology education leading to assumptions about how it is taught in secondary and how some teaching topics should be transferred within the primary curriculum. This indicates that there could be even greater differences in understanding than the higher-level quantitative data suggests.

Through the data, problem solving was recognised as important, predominantly by secondary educators (Table 1). Respondents from both sectors, however, suggested that they utilise problem solving pedagogical approaches in some way, where 64% of secondary and 58% of primary incorporate this element into their regular teaching of D&T (Table 3). This suggests that there can be similar approaches in key areas of technology education, although not always directly recognised through the given definitions. Perhaps this further illustrates the concept that some teachers do not hold a secure understanding of technology education (Education Scotland, 2014) and the core characteristics the subject possesses in Scottish schools.

### Interdisciplinary learning and topic teaching around impact on society and application of engineering

Considering the definition of D&T education using the combined coded results from the initial open-ended question (Table 1) the area identified most frequently (by a total of 14 primary and secondary participants) was the ‘impact on society’. Both primary (5 participants) and secondary participants (9 participants) recognised that a real-world application was central to the subject. The Stage 1 quantitative questionnaire data showed further agreement between teachers in each sector. When asked about ‘topics involving real world scenarios and society’ nearly half of primary (42%) and secondary (48%) teachers (Table 3) responded that this featured ‘often’ in their teaching.

The importance of real-world application of D&T teaching was further supported where the CfE ‘application of engineering’ outcome was identified as something taught by most primary teachers (53%) and by a high proportion of secondary practitioners (Table 2). However, in the initial open-ended question teachers at secondary level rarely referred specifically to engineering, although it was recognised by *one* participant who suggested that technology education was:

the understanding and application of engineering and science principle[s] to solve everyday problems. (Secondary teacher)

This does not mean that secondary teachers are ignoring engineering as a part of D&T education, as many of the definitions could be interpreted to embed the principles of engineering without explicit reference. Furthermore, these practitioners associate design and creative approaches in their teaching, and this supplements the objective, technical knowledge that engineering is renowned for (Wilson, 1993). This finding draws attention to this area of the curriculum and suggests a valuable opportunity for greater cross-sector collaboration, using engineering contexts as a vehicle. This could have significant benefits as engineering knowledge and skills are considered to drive and support future growth in Scotland’s economic and innovation developments (SEEAG, 2012).

Overall, this suggests that both primary and secondary teachers consider links between D&T teaching to industry, and relevant sectors or professions related to engineering. The identification of this common or shared understanding may be due to the obvious practical nature of D&T teaching and this could provide a starting point for building greater depth and breadth in common understanding.

### Practical and technological skills

The initial question, which asked participants to consider how they would define D&T teaching, resulted in respondents referring to both practical and technological skills (Table 1). Practical skills were referenced by equal numbers of primary and secondary participants (n = 2) whereas technological skills were more frequently referenced by secondary (n = 7) than primary teachers (n = 2).

Many secondary educators identify the requirements of practical skills through their definitions; demonstrating the substantial influence this element has within the secondary curriculum. The findings show that 68% of secondary teachers *often* offer design and construct themed projects whilst 86% engage learners through practical pedagogical approaches (see Tables 2 and 3). This is illustrated by one secondary participant who responded saying; ‘*It is a practical and knowledge-based subject’* (Secondary Teacher). Furthermore, data from the Stage 2 interviews confirm the importance of this element for all secondary educators due to the requirement of pupils to engage in practical projects.

In comparison a smaller percentage of primary educators openly recognised practical skills as an area that they cannot offer to their pupils. Just under half (47%) indicated they do not use any practical pedagogical approaches (Table 3). One reason may be that primary educators associate technology with computing or digital technology. It could also be related to primary practitioner’s interpretation of curriculum guidance where practical skills are associated with being in a workshop, as they themselves may have experienced in school. Despite this lower level of recognition from primary practitioners some were able to see beyond technology being digitally focussed, as one participant explained:

Technology education is about learning about technology systems including practical technologies (Primary Teacher).

In addition, both primary interviewees expressed concerns regarding their ability to offer such experiences, which may provide some explanation for the lack of recognition of this element.

### Teaching use of technology versus understanding technology

This final theme may explain the differences between the primary and secondary participants, which is based on a fundamental different understanding of the subject of D&T. The primary teachers in this study were far more likely to consider the D&T subject as being about how to *use* technology, in particular digital technology, rather than deepening understanding about wider forms of technology. The data from the primary sector participants revealed a high proportion referring to either their own, or their pupils’ use of technology to support learning and teaching. To illustrate, one respondent described technology education as:

a valuable tool that can be used to enhance education in many positive ways if used appropriately. (Primary Teacher).

Another participant reported the relevance of:

teaching children how to use the tools at their disposal, e.g., computers. (Primary Teacher).

Although the use of technology to support teaching and learning was a common thread amongst primary teachers, for some this was also supplemented by reference to other elements such as practical application or problem solving:

technology education is about making stuff and exploring problems. Using digital technology to aid learning (Primary Teacher).

Reasonably, this could indicate that primary teachers are aware of other elements of technology education yet the high recognition of the *use* of technology to aid learning is difficult to ignore. It appears that, in some instances, the boundaries have become blurred between the understanding of digital technologies and the D&T curriculum area demonstrating that some primary educators associate their understanding of technology education with the use of digital devices. Furthermore, this possibly reveals less of an understanding of D&T as a subject and represents the broader nature of the CfE Technology Curriculum (Education Scotland, no date).

### Primary and secondary educator knowledge of other sector and stage of education

The final general theme centres on how participant teachers from each sector view their counterparts in the other stage or sector of education. This was informed by a question in the Stage 2 interviews where participants were asked what they knew about their respective learning stage. All educators discussed knowing very little about D&T learning in primary or secondary. A secondary teacher identifies that they ‘*should be more up to speed’* with primary learning while another secondary teacher acknowledged:

I know nothing [about the primary curriculum] other than the conversations I’ve had with primary teachers about it. What they’ve said is that they’re not confident to deliver it at all and therefore don’t deliver it. (Secondary Teacher)

One primary teacher discussed their knowledge of secondary D&T through their experience as a secondary school teacher whereas another primary teacher admitted to knowing ‘*honestly, nothing’.* It can be concluded that, from this small sample of teachers, they have limited knowledge or experience about the other’s approach in D&T. This deficiency of their respective primary or secondary D&T knowledge must be emphasised as a concern and an obvious barrier to continuous learning in the BGE. Therefore, opportunities to discover more about respective learning stages should be sought. Davies and McMahon (2011), for example, discuss colleague observation and pupil portfolios as effective methods of learning about the other and connecting experiences. Collaborative activities can also support the sharing of good practice and knowledge, which is valuable in a specialist subject such as D&T.

The ambition to create effective collaborative links across schools seems to be straight forward through this research discussion, though the high numbers of practitioners who do not collaborate put the reality of time, workload barriers and collaboration opportunities into context. Participating educators identified similar barriers to embedding transition projects into their planning, namely time and staffing. However, the need for a shared understanding and approach to D&T education remains. Perhaps school communities need to place collaboration and coherency higher up on the agenda to enhance young people’s academic progression and overall attainment. As Donaldson (2010) promotes, teachers are the leading players in shaping and affecting educational change fit for 21st century learners therefore, teachers are at the forefront of creating coherent approaches across D&T learning.

## Conclusion

This research has shown that, with respect to curricular subject definition and teaching approaches, the shared knowledge and understanding of Design and Technology across primary and secondary sectors in Scotland is an issue. Curriculum for Excellence was designed to create a more coherent learning experience however the results of this research project shows that the reality does not represent the policy rhetoric. Therefore, if there is to be a coherent curricular experience for learners moving from primary into secondary education, and beyond into STEM careers, then there needs to be a focus on concrete actions.

Although participants identified some common themes of technology through their understanding and areas of teaching, it does not determine that there is a shared approach, therefore continuity and coherency of learning cannot be guaranteed. This is best illustrated with the example of teaching the design process. If the design process is taught through a sequential approach (at the primary stage) and then later (at the secondary stage) using a mixed approach; there may be confusion for learners in their understanding, affecting their independence of learning and disrupting their progress. This may be particularly applicable to learners who already find the concept of designing a challenge. Even though both sequential and mixed approaches may be suitable, practitioners need to be mindful of the learning that has come before and therefore adapt, communicate, and justify the learning that will come next (Growney, 2013). For senior stage (secondary school) learners who have more design experience, such an adjustment may act as a learning challenge, whereas for learners involved in the primary to secondary transition this change in approach may be harmful to their progression (Williams, 1997; Braund, 2008).

The current study revealed, more specifically, that the underpinning issue may stem from differences in teacher definitions of technology education, which may then lead to different approaches to the subject, across the two sectors. Drawing on the work of Braund (2008), these findings are important as such disparities will disrupt learning in the subject; thereby further affecting learner’s progression and attainment. Despite this difference, primary and secondary teachers shared some similarities including the recognition of designing and the inclusion of problem solving and to a lesser extent practical work. What is less clear, from the current study, is depth to which any primary practitioners may misunderstand the specialist concepts within D&T education, and this may provide the basis for future research.

### Recommendations

This research project set out to better understand coherent approaches within the technologies curricular area within the Broad General Education (BGE) stage of CfE in Scotland. In particular, the main objective was to determine teacher’s understanding of technology education by considering primary and secondary educators’ shared definitions and approaches to teaching D&T and considering the implications of this. Although the primary research that informed this study determined that creating truly coherent learning experiences was difficult for schools to achieve, giving an absolute answer to this question has not been possible. The research does propose, however, the concept that mixed approaches to learning do exist, as well as notable differences in teacher definitions of technology education that should be addressed. Through the staircase curriculum model, Au and Raphael (2011, Fig. 1) emphasise the outcomes of delayed progression and the inability to reach academic potential.

These findings discussed should be used as a starting point for critical reflection and professional discussion with the aim of maximising learner’s achievement and progression in D&T education. This could be achieved through greater emphasis on collaboration and shared, cross-sector working. One way to achieve this may be a focus on transition stages by teachers in each sector. An alternative may be to consider the role of specialists for D&T within the primary sector. Ultimately, however, for these initiatives to succeed there must also be commitment from the practitioners themselves and so a focus on attitudinal, and intellectual elements of teacher professional development and learning (Evans, 2014), and teacher agency (Priestley et al., 2015) will be fundamental to enacting any curriculum change.

The research presented has shown that although Education Scotland (no date) provides educators with the tools to create continuous learning pathways, more needs to be done across school communities in realising this goal. Fundamentally, school communities should prioritise time and resources for shared D&T planning and teaching (Au and Raphael, 2011). For D&T education to be coherent and progressive, educators must plan learning that builds upon the learner’s previous experience, avoiding repetition, whilst also having a secure understanding of where the learning will go next. At the heart of this is open communication between primary and secondary schools that can be sustained across learning stages. To realise this, teachers need to move beyond seeing the two sectors separately. Educators must recognise that there is a lot to gain from others, where partnerships should be established through respect and trust (Growney, 2013).

In building partnerships, Davies and McMahon (2011) recommend a range of approaches including: co-observations of teaching, improving knowledge, shared assessment and jointly planned teaching. Collaborative networks must run deeper than one-off transition projects, where continuous learning must incorporate coherence across curriculum understanding and pedagogical approaches (Braund and Hames, 2011). However, this would be a naïve recommendation without considering the time and commitment for teachers such an endeavour would take to achieve; essentially becoming the greatest barrier to continuous learning. Recognising the pressure on resources, including time, Donaldson (2010) emphasises the enhanced use of partnerships by advocating that school communities should ‘make the most of what we already have’ (Donaldson, 2010, p. 2).

At practical school organisation level, difficulties lie where the structure of technology BGE differs between primary and secondary schools. At primary level, the field is dispersed amongst other areas of the curriculum whilst secondary learning narrows down into distinct, specialist subject areas. Continuing in this manner is always going to support a divide however through collaboration, teachers must aim to minimise disruption to curriculum, pedagogical and assessment differences to maximise learning. For primary educators who teach across the whole curriculum, making connections with every subject specialism at secondary level would be unrealistic. Therefore, the potential for schools to support primary teachers to become subject specialists, with increased support from their secondary colleagues, needs to be realised.

Educators must act to improve their communication between schools and work towards a shared understanding of D&T concepts. Ultimately, teachers should endeavour to enhance learning experiences by reflecting upon their own understanding of technology education and the ways in which they deliver this to pupils; actively considering prior learning and gaining knowledge of future pathways. As Donaldson (2010) articulates, through collaboration and support, teachers should ‘take responsibility for their own professional development, building their pedagogical expertise, [and] engaging with the need for change’ (p. 84). This professional expectation places practitioners as key contributors in shaping learning and affecting change within the D&T curriculum.
